# Does scale matter? A systematic review of incorporating biological realism when predicting changes in species distributions

**DOI:** 10.1371/journal.pone.0194650

**Published:** 2018-04-13

**Authors:** Sydne Record, Angela Strecker, Mao-Ning Tuanmu, Lydia Beaudrot, Phoebe Zarnetske, Jonathan Belmaker, Beth Gerstner

**Affiliations:** 1 Department of Biology, Bryn Mawr College, Bryn Mawr, Pennsylvania, United States of America; 2 Department of Environmental Science and Management, Portland State University, Portland, Oregon, United States of America; 3 Biodiversity Research Center, Academia Sinica, Taipei, Taiwan; 4 Department of Ecology and Evolutionary Biology and Michigan Society of Fellows, University of Michigan, Ann Arbor, Michigan, United States of America; 5 Department of Forestry, Michigan State University, East Lansing, Michigan, United States of America; 6 Ecology, Evolutionary Biology, and Behavior Program, Michigan State University, East Lansing, Michigan, United States of America; 7 School of Zoology and the Steinhardt Museum of Natural History, Tel Aviv University, Tel Aviv, Israel; 8 Department of Fisheries and Wildlife, Michigan State University, East Lansing, Michigan, United States of America; Universita degli Studi di Napoli Federico II, ITALY

## Abstract

**Background:**

There is ample evidence that biotic factors, such as biotic interactions and dispersal capacity, can affect species distributions and influence species’ responses to climate change. However, little is known about how these factors affect predictions from species distribution models (SDMs) with respect to spatial grain and extent of the models.

**Objectives:**

Understanding how spatial scale influences the effects of biological processes in SDMs is important because SDMs are one of the primary tools used by conservation biologists to assess biodiversity impacts of climate change.

**Data sources and study eligibility criteria:**

We systematically reviewed SDM studies published from 2003–2015 using ISI Web of Science searches to: (1) determine the current state and key knowledge gaps of SDMs that incorporate biotic interactions and dispersal; and (2) understand how choice of spatial scale may alter the influence of biological processes on SDM predictions.

**Synthesis methods and limitations:**

We used linear mixed effects models to examine how predictions from SDMs changed in response to the effects of spatial scale, dispersal, and biotic interactions.

**Results:**

There were important biases in studies including an emphasis on terrestrial ecosystems in northern latitudes and little representation of aquatic ecosystems. Our results suggest that neither spatial extent nor grain influence projected climate-induced changes in species ranges when SDMs include dispersal or biotic interactions.

**Conclusions:**

We identified several knowledge gaps and suggest that SDM studies forecasting the effects of climate change should: 1) address broader ranges of taxa and locations; and 1) report the grain size, extent, and results with and without biological complexity. The spatial scale of analysis in SDMs did not affect estimates of projected range shifts with dispersal and biotic interactions. However, the lack of reporting on results with and without biological complexity precluded many studies from our analysis.

## Introduction

Climate change already has and will continue to alter environmental conditions in increasingly severe ways [1,2]. With changing environmental conditions, species must adapt, migrate, or face extirpation. Our ability to predict such changes is critical to conservation efforts. Species distribution models (SDMs) have become the conventional approach for modeling the current and future geographic distributions of species [3–5]. The most widely used SDM approaches are problematic in that they rely largely on examining correlative relationships between abiotic drivers and species occurrence data to predict future changes in distributions while ignoring important aspects of species’ biology [6–10]. In particular, biotic interactions, dispersal ability, and interactions between the two can greatly influence a species’ range [8,9,11–13]. For instance, vagile butterflies may not colonize climatically suitable habitat as it becomes available because they depend on dispersal limited host plants [14].

Scaling paradigms in ecology suggest that influences of abiotic and biotic processes on species distributions vary across grains and extents [15–17]. For instance, climate is expected to have the largest influence on species distributions at coarse (i.e., continental) and fine (i.e., local microclimatic) grains. Biotic interactions are assumed to have only local effects on species distributions, whereas dispersal is expected to play an important role in determining species distributions at local or global spatial scales, while effects of dispersal at intermediate spatial scales remain vague [17]. Recent studies show how the influence of dispersal and biotic interactions on species distributions can depend upon spatial scale (i.e., grain of the data and extent of the study area); [18–21]. However, there has been little synthesis of the literature to elucidate how the choice of specific spatial scales used in SDMs may influence model outputs when dispersal, biotic interactions, or interactions between the two are incorporated.

**Fig 1:**
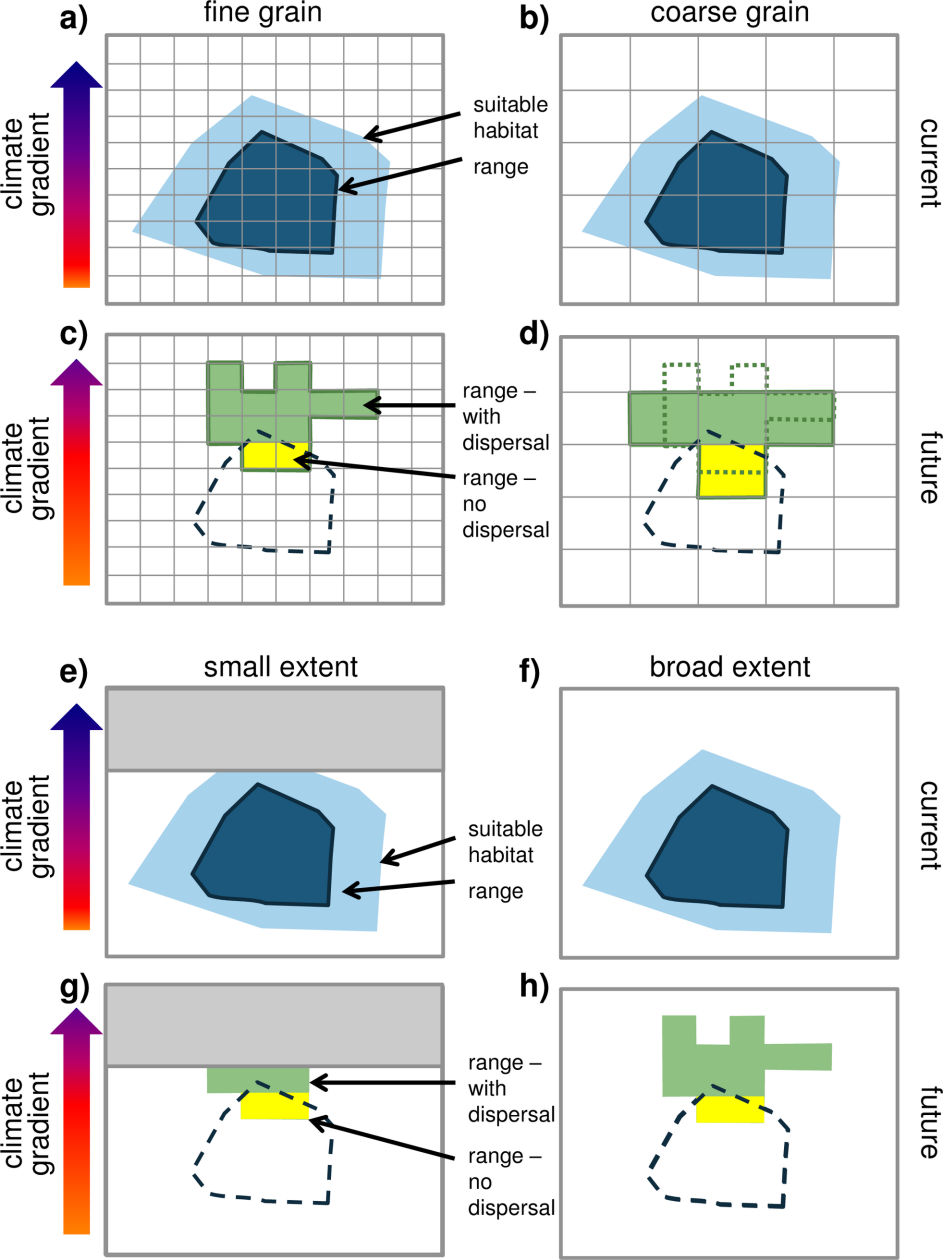
Conceptual diagram of how spatial grain size (a-d) and extent (e-h) could affect the interpretation of species range shifts using species distribution models. A species current range (inner dark blue object) and suitable habitat (outer light blue object) are shown for a fine (a) and coarse (b) grain size. Future suitable habitat (outer green solid line) is shown with two scenarios, no dispersal (yellow shading) and with dispersal (green shading) for fine (c) and coarse (d) grains. For illustration, the current range is shown with dashed lines in the bottom panels, and the suitable future habitat from the fine grain size is superimposed on the coarse grain size future scenario. In these illustrations, for a cell to be counted as containing the species, at least 50% of the cell must be occupied. In the fine grain example, the species range is reduced by 86% for no dispersal and 14% with dispersal, compared to 67% loss of range size for the no dispersal scenario in the coarse grain example and no loss in range size with dispersal. In this particular example, coarse grain size underestimates the loss of range size for species. However, many studies make the assumption that if a species occupies any fraction of a grid cell, then it is considered present. With this assumption, coarser grain sizes would still underestimate range loss with climate change (fine grain: no dispersal = -90%, dispersal = -43%; coarse grain: no dispersal = -86%, dispersal = -14%). In contrast, a study with a small, localized extent (e, g) could conceivably underestimate species range shifts compared to a study with a broad extent (f, h).

Understanding how spatial scale interacts with biological processes to influence species distributions and their responses to changes in climate is a fundamental question of biogeography with implications for conservation biology and global change biology. As greater biological complexity is incorporated into SDMs, it is important to consider spatial scale because the grain and extent of species and environmental data are likely to affect the resulting predictions, which are often used for generating conservation plans and climate change policy pertaining to biological diversity [22–26]. Species distribution models may show scale dependence either because of the scaling of biological processes or because of statistical scale dependence. For an illustration of statistical scale dependence, we show conceptual scenarios of how spatial grain size (Fig 1) and spatial extent (Fig 1) influence the prediction of changes in a species’ range sizes. For instance, if a local extent excludes some of a species’ suitable habitat, then even with full dispersal to any area with suitable habitat the predicted range of the species will be underestimated by a model fit with the local extent compared to a model fit with a broader extent that encompasses all suitable habitat (Fig 1).

Recent works provide comprehensive reviews of how modelers incorporate dispersal and biotic interactions into SDMs with approaches ranging from very simple to complex, with more sophisticated approaches limited by data availability [8,9,27–30]. For instance, simplified implementations of dispersal in SDMs consider no dispersal vs. full dispersal (i.e., a species is capable of reaching any suitable habitat), whereas more complex approaches incorporate migration time lags or taxon-specific dispersal capacities [27,29]. A basic approach to incorporating biotic interactions into SDMs is to include the distribution of species X as an independent variable along with abiotic independent variables to predict the distribution of species Y (e.g., [6]), whereas a more complex approach links SDMs of interacting species to dynamic process models accounting for population dynamics and dispersal (e.g.,[31]).

Despite recent reviews on model implementation, there is no current synthesis of whether scale affects model predictions when biotic interactions or dispersal are included, and if so, what spatial scales are needed to model biotic interactions and dispersal. The first objective of this paper is to determine the current state of SDMs incorporating biotic interactions and dispersal by identifying key knowledge gaps in the literature. Specifically, we examine the spatial scales at which such SDMs have been implemented in the context of where studies have been conducted geographically, which ecosystems have been studied, which species have been modeled, and the usage of climate models. Our second goal is to understand how the scaling of biological processes changes SDM predictions.

## Materials and methods

### Data compilation

**Fig 2:**
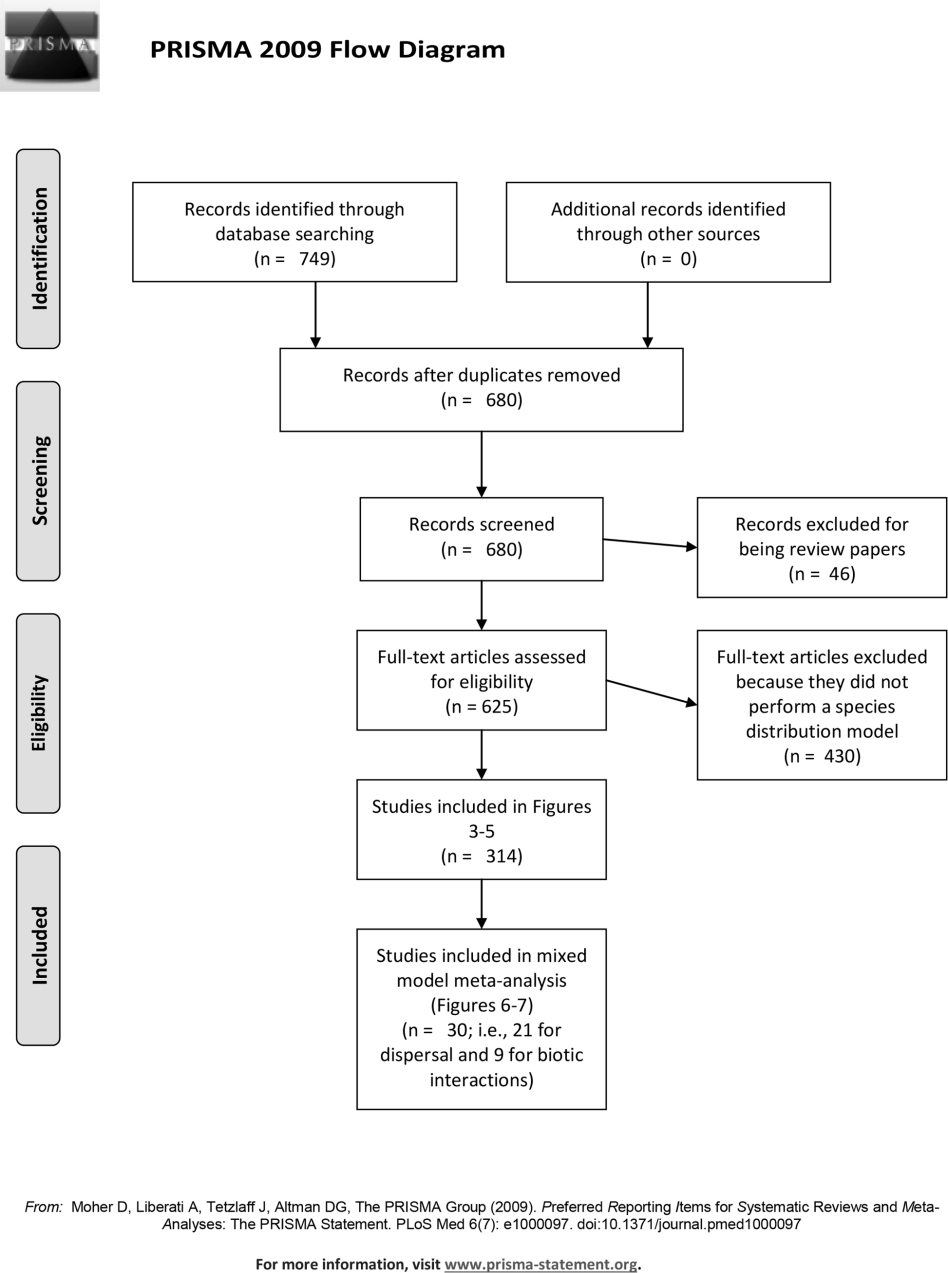
Preferred reporting items for systematic review and meta-analyses (PRISMA) flow chart of steps taken in the systematic review process that document the sample size (n) of journal articles at each step [31].

We conducted three ISI Web of Science searches to identify the pool of relevant papers. All searches included “climate change”, “predict*”, “model*”, “species” and “distribution OR range” as topics. The first search included the additional topic “dispers*”, the second search included “biotic interact*” and the third search included both “dispers*” and “biotic interact*”. All ISI Web of Science searches included papers published and indexed between 2003 and 2015. In our review, we included papers that employed SDMs to model geographic range sizes (Fig 2 and S1 File).

In order to determine how the scale dependence of biological processes changes species distribution predictions, we compiled data on spatial scale (grain and extent), biotic interactions, and dispersal for each relevant publication (S1 Table and S2 File). The extent of a SDM was considered regional if it did not span an entire continent and continental if it did. Papers were counted as including biotic interactions if they specifically modeled competition, facilitation, predation, parasitism, mutualism, amensalism, commensalism, or antagonism. We also compiled additional data on: the taxa modeled, location of the study, whether and how many multiple general circulation models were projected to a time point other than current (S1 Table).

### Quantitative data analysis

Of the 680 full text papers that our search criteria generated, 314 papers were considered appropriate for our study objectives (Fig 2). Of these 314 papers, 134 incorporated dispersal into the SDMs, 56 incorporated biotic interactions, and 25 incorporated both biotic interactions and dispersal into the same SDM (S1 and S2 Files). Only a subset of the papers comparing models with and without dispersal and biotic interactions provided numerical data on projected range size that would be suitable for further analysis– 21 papers for dispersal, 9 papers for biotic interactions, and none for both dispersal and biotic interactions (S2 and S3 Files). When the spatial extent of these models was not reported in papers, we estimated it based on the description and/or map of the study area boundaries. All but six of the 21 dispersal papers included in this analysis incorporated dispersal by assuming ‘full’ dispersal in which organisms were capable of filling in the entire projected niche, so we only compared ‘full’ vs. ‘no’ dispersal (i.e., organisms were not able to fill the projected suitable areas beyond their current ranges) in the further analysis. For the papers that incorporated dispersal in realistic ways, only the ‘full’ dispersal scenario was included in the analysis described below. Thus, the final sample size for dispersal studies was *n* = 21 and for biotic interactions was *n* = 9 (S3 File).

To quantify the effect of incorporating biological processes in SDMs on the projected changes in species distribution ranges under changing climates (objective 2), we calculated the percent range size changes for models with and without dispersal and for models with and without biotic interactions for each species modeled within a study. We then calculated an effect size of incorporating biological processes as:

$$EffectSize=\frac{CR_{included}-CR_{excluded}}{\left|CR_{excluded}\right|}\times 100$$ (1)

Where *CR*_*included*_ is the change in range size projected by a model with either dispersal or biotic interactions included in the SDM and *CR*_*excluded*_ is the change in range size projected by a model with either dispersal or biotic interactions excluded for the same species.

To explore how spatial extent and grain size of a model influence the effect size, we built linear mixed models with effect size as the response variable and the extent and/or grain size as fixed-effects variables. The predictor variables were log_10_-transformed before fitting the models to meet the assumptions of the Gaussian distribution of residuals. Spatial extent and grain covaried moderately for dispersal (ρ = 0.02 for untransformed values; ρ = 0.56 for log10 transformed values), so we tested for the two fixed effects additively in a single mixed effects model. We also calculated variance inflation factors (V.I.F.’s) to confirm that the spatial grain and extent did not co-vary in the mixed effects model. For the dispersal mixed model, variance inflation factors indicated that additive effects (but not, higher order interactive effects) of grain and extent were not correlated (V.I.F.’s ~1.5 for both), so we excluded the interaction term between the two. However, extent and grain varied significantly for biotic interactions (ρ = 0.71 for untransformed values; ρ = 0.73 for log10 transformed values), therefore the effect of these variables on effect sizes were tested separately.

Individual species modeled within a paper were treated as nested replicates within the linear mixed models, so a term indicating the manuscript to which a species belonged was included in the models as a random effect. For studies that projected species ranges to multiple future time periods, we included the average response across time periods. For studies that projected species range changes under multiple emissions scenarios and/or general circulation models for a single future time period, we included the average response across emissions scenarios and/or general circulation models. We weighted the effect size response of the mixed effects models by the change in climate predictors between current and future values. Several papers reported the absolute change in mean annual temperature and percent change in mean annual precipitation between current and future values. For those papers that did not report these changes in temperature and precipitation, we downloaded the raw climate data and calculated them for the spatial extent of the study. We note that focusing on mean precipitation and temperature values may not capture the exact predictor variables used in each study, but this provides a rough idea of change in climate space for each study in a standardized manner. Changes in temperature and precipitation across studies for dispersal or biotic interactions data sets were standardized across studies to range between zero (no change between current and future climate) and one (maximal change between current and future climate) because temperature and precipitation changes were in different units. The sum of these standardized values for changes in temperature and precipitation for each study were used as weights in the mixed effects model (S3 File). To account for variability in the response due to taxonomic differences, the taxonomic group under study was also included as a random effect. Thus, the final sample size for dispersal studies was *n* = 510 and for biotic interactions was *n* = 24. Complete details on the number of replicates nested within each study and taxonomic group are available in S3 File. We include a PRISMA checklist for our systematic review (S2 Table).

## Results

**Fig 3:**
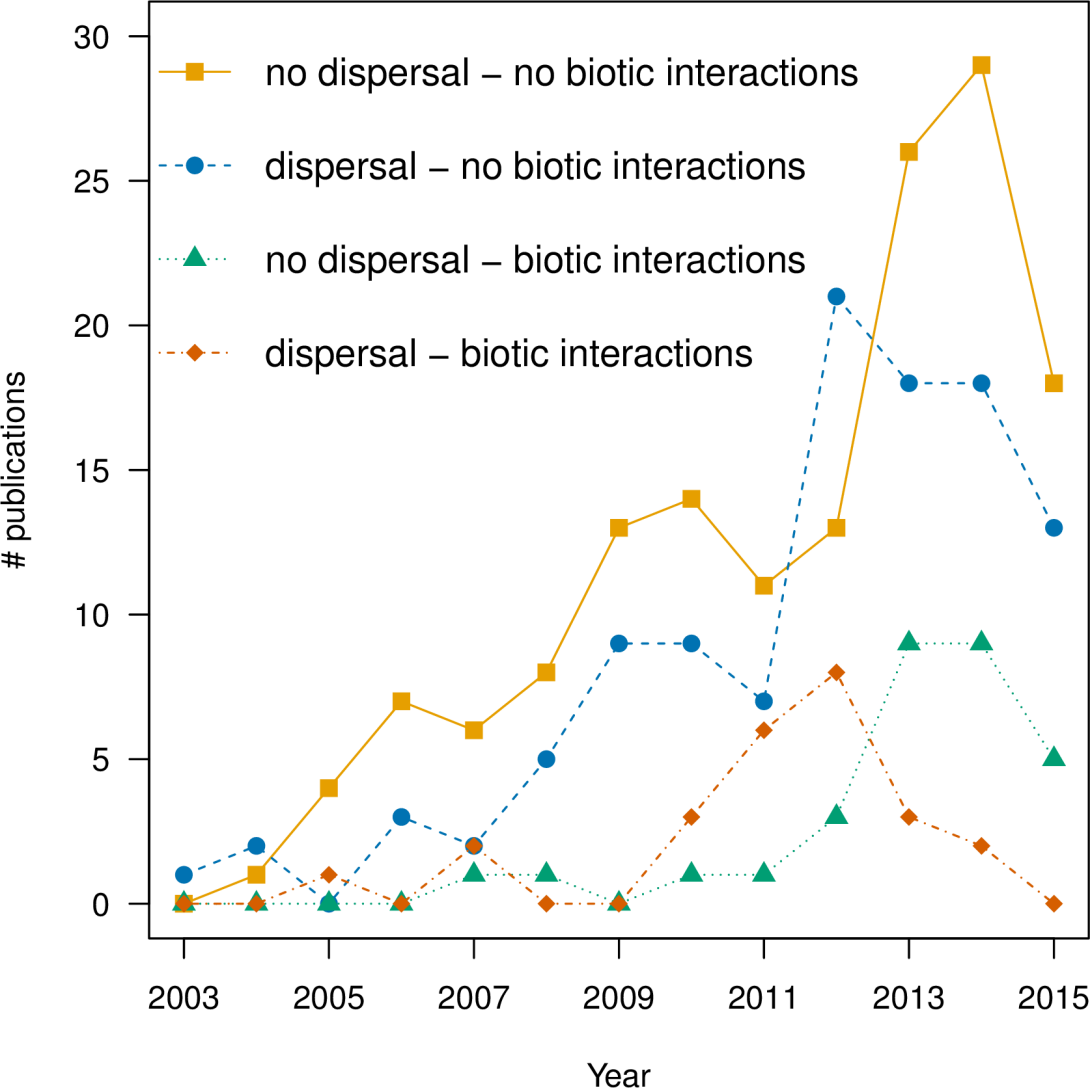
Number of publications modeling future species distributions from 2003–2015. Publications were categorized by the inclusion (or lack thereof) of dispersal and biotic interactions.

Our three ISI Web of Science searches yielded 680 papers after removing duplicate records, with 314 deemed as appropriate for our study. See Fig 2 for a flow chart of sample sizes of papers during the screening and eligibility steps of the systematic review leading up to the final sample sizes of studies included in the analyses [32]. There has been a consistent increase in the total number of publications using SDMs, from a single paper in 2003 to >58 in 2014 (with a slight dip in 2015) (Fig 3). Additionally, there have been steady increases in the numbers of studies that included dispersal (134 cumulative studies), biotic interactions (56 cumulative studies), or both (25 cumulative studies) through 2011 and 2012 (Fig 3). However, those studies incorporating biotic interactions and dispersal drop thereafter. Less than half (49%) of the publications that incorporated dispersal used biologically-relevant dispersal, such as taxon-specific rates, with the majority (74%) occurring since 2012.

**Fig 4:**
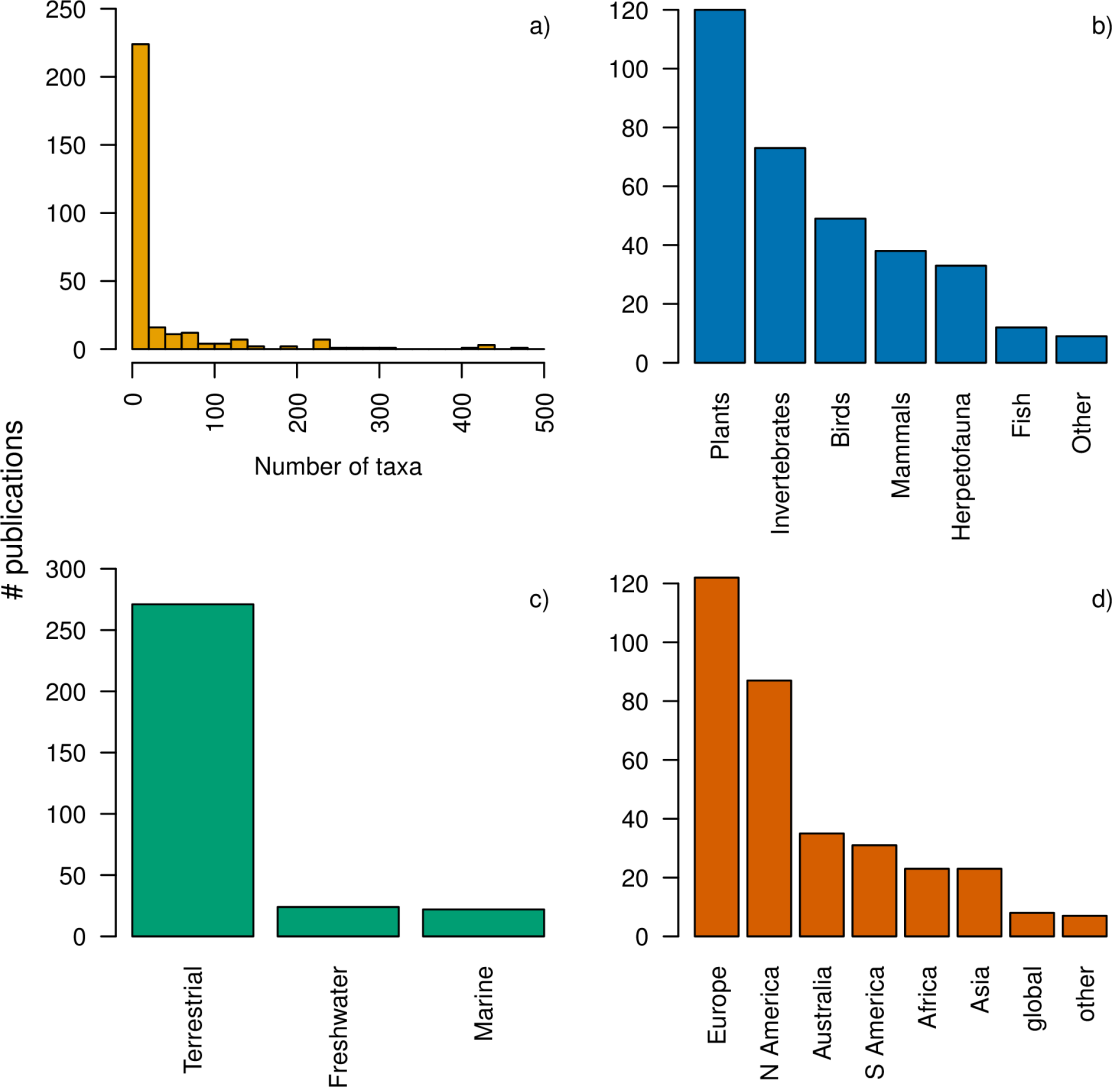
a) Number of taxa, b) type of taxa, c) realm and d) continent considered in relevant publications. Note that the bars in each panel will not necessarily sum to the total (*n* = 314), as some studies were conducted on multiple taxa or continents, or were excluded as they could not be easily assigned to one category (e.g., oceans).

**Fig 5:**
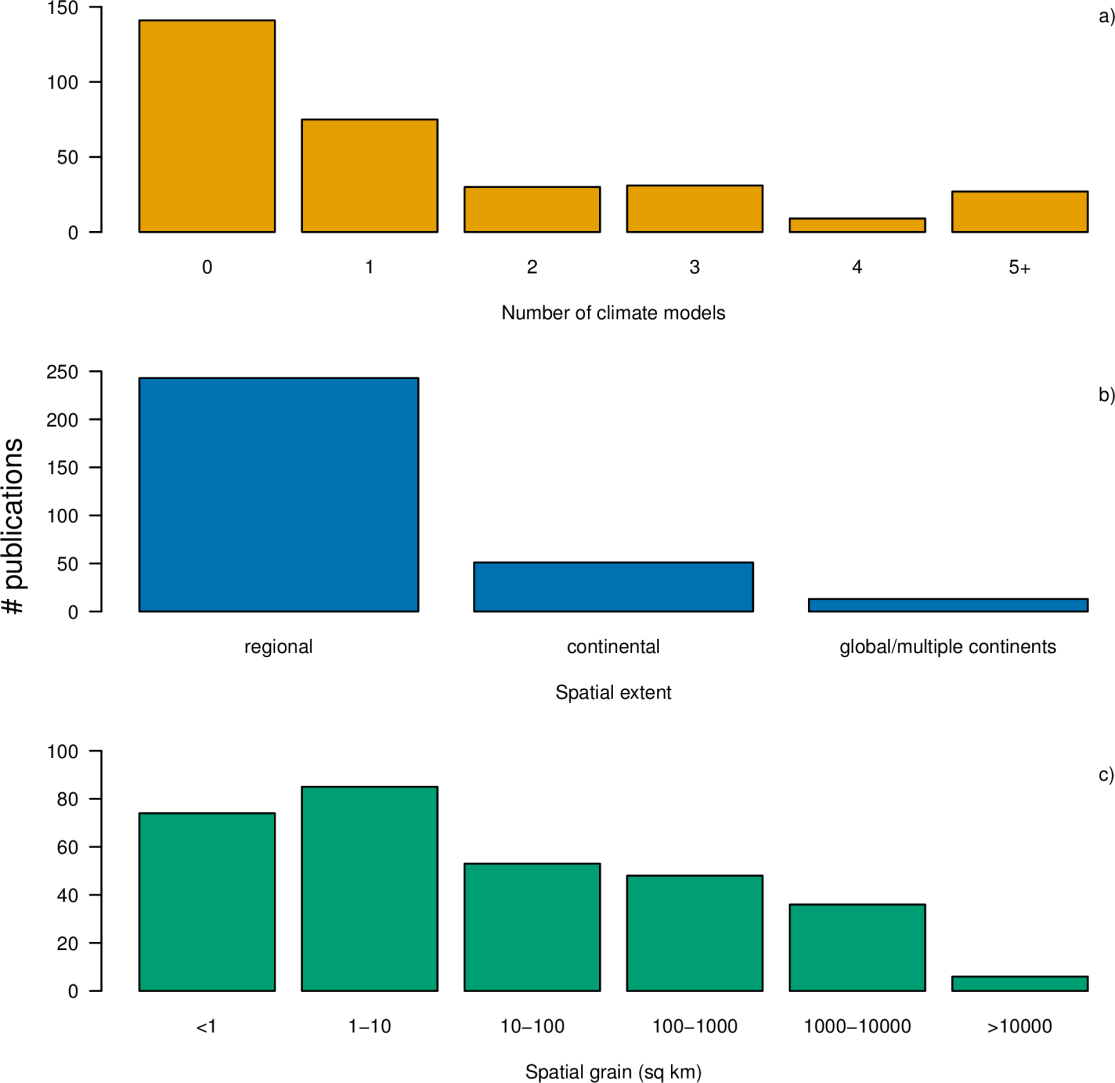
a) Number of climate models, b) model spatial extent, and c) model spatial grain (sq km) for the relevant papers in this study. Note that the bars in each panel will not necessarily sum to the total (*n* = 314), as some studies were conducted at multiple spatial extents or grains, or were excluded for lack of reporting.

The majority of the 314 relevant studies focused on a small number of species (Fig 4). An additional 16 studies not shown in the histogram included >500 species (range 527–11012 species). Studies largely focused on plants followed by invertebrates, and vertebrates more generally (Fig 4), and almost all of the studies were from the terrestrial realm (Fig 4). Approximately 39% of the studies were from Europe and the fewest studies were from Africa and Asia (Fig 4). About 45% of SDM papers did not project the model fits into the future, and most of those SDM papers that did project into the future only modeled one future climate scenario (44%; Fig 5). Most SDMs were regional in spatial extent (79%; Fig 5) and performed at spatial grains of <10 km^2^ (53%) or 100–10,000 km^2^ (28%; Fig 5C). Of those papers that reported both spatial grain and spatial extent, there was not a strong correlation between the two (ρ = 0.34–0.53 depending on whether two outlier studies–one with high spatial grain and one with high spatial extent were included; S1 Fig).

**Fig 6:**
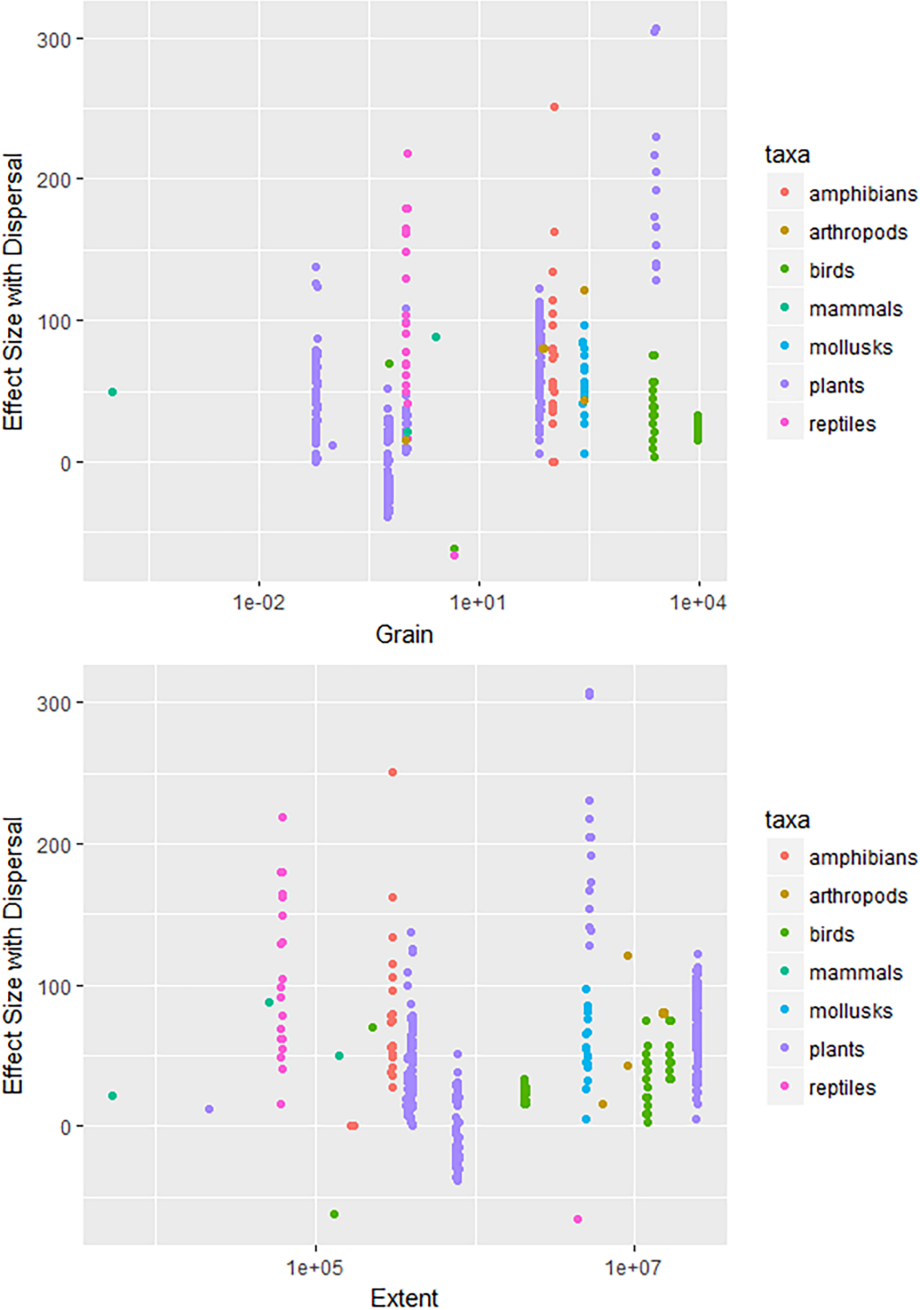
Effect sizes for changes in range size weighted by change in climate space, from species distribution models with vs. without dispersal by a) grain and b) extent for each taxa. Effect sizes are calculated as in Eq 1. Higher effect sizes indicate larger influences of incorporating dispersal in a model on projected range changes.

**Fig 7:**
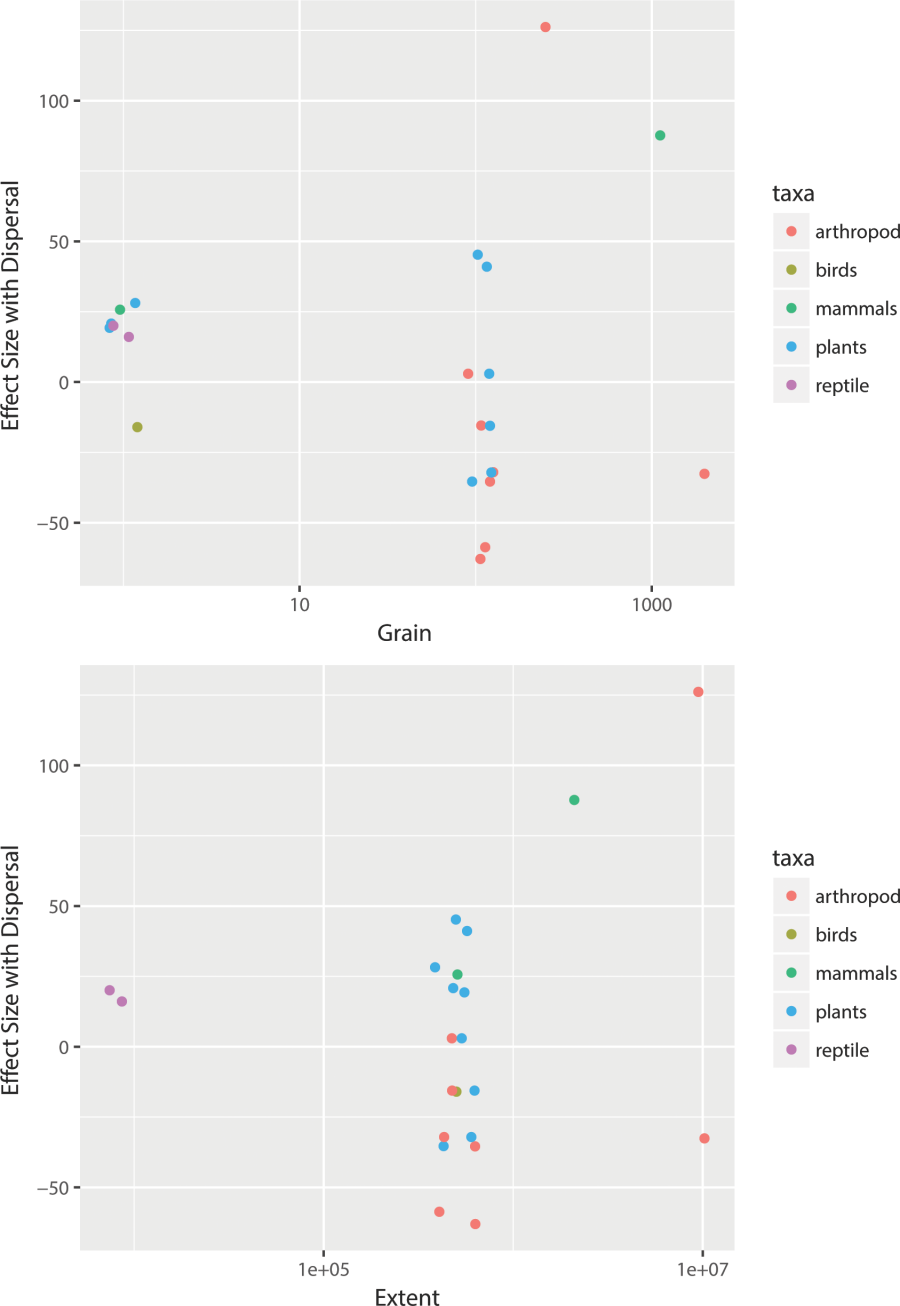
Effect size for changes in range size weighted by change in climate space, from species distribution models with vs. without biotic interactions by a) grain and b) extent for each taxa. Effect sizes calculated as in Fig 6. In a), all points plotted at ~100 for plants and arthropods are from Romo et al. [33] and indicate butterflies and their host plants. In b), all points coded as plants or arthropods at intermediate extents, except for three plants with effect sizes around 25, refer to the Romo et al. [33] host plants and butterflies.

Limited reporting on differences in SDM predictions with and without biotic interactions and/or dispersal precluded most studies from the effect size analysis. There were neither significant effects of spatial grain or spatial extent on the effect sizes for dispersal for projected changes in range sizes (Table 1 and Fig 6). Effect sizes for dispersal for projected changes in species range sizes did not show clear differences among taxa with different levels of mobility (Fig 6). For instance, immobile plants exhibited both low and high effect size response ratios. In general, the effect sizes for biotic interactions were small (Fig 7), compared to effect sizes for dispersal. There were no significant effects of spatial grain or spatial extent on effect sizes for models with biotic interactions (Tables 2 and 3 and Fig 7).

**Table 1:** Analysis of deviance table (Type III Wald χ^2^ test) from linear mixed model testing the influence of spatial grain and extent on the effect size of incorporating dispersal for projected changes in species range size.

Parameter	χ^2^	df	*P*
Intercept	23.13	1	<0.001
Log_10_(Grain)	0.97	1	0.34
Log_10_(Extent)*	2.32	1	0.13

Effect sizes were weighted by change in climate space between current and future values.

**Table 2:** Analysis of deviance table (Type III Wald χ^2^ test) from linear mixed model testing the influence of spatial grain on the effect size of incorporating biotic interactions for projected changes in species range size.

Parameter	χ^2^	df	*P*
Intercept	1.33	1	0.27
Log_10_(Grain)	0.31	1	0.60

Effect sizes were weighted by change in climate space between current and future values.

**Table 3:** Analysis of deviance table (Type III Wald χ^2^ test) from linear mixed model testing the influence of spatial extent on the effect size of incorporating biotic interactions for projected changes in species range size.

Parameter	χ^2^	df	*P*
Intercept	1.39	1	0.26
Log_10_(Extent)	0.66	1	0.45

Effect sizes were weighted by change in climate space between current and future values.

## Discussion

There is wide interest in moving beyond SDMs based solely on relationships between abiotic factors and occurrence data to models that more meaningfully incorporate biological processes [6–10,16]. Despite growth in the number of studies incorporating greater biological realism into SDMs in the last decade (Fig 2), our review and analysis identified key knowledge gaps in our understanding of the importance of biotic complexity and spatial scale in modeling future species distributions.

Approaches to incorporating dispersal into SDMs in climate change-related studies vary in complexity [28,29]. Our review suggests that while more papers are including dispersal into SDMs in climate change-related studies, the approaches taken to incorporating dispersal into SDMs are largely simplistic (e.g., assuming that a species is able to fully track changes in available habitat). Specifically, the inclusion of dispersal rates that account for taxon-specific differences in dispersal ability was minimal moderate (49%), though this largely reflects changes in the last four years of our dataset: prior to 2012, only 14% of studies incorporated taxon-specific dispersal rates (29%). There is ample evidence that species differ greatly in dispersal abilities (e.g., [19,34,35]), and these differences in dispersal capacities may influence their ability to track suitable habitat in the face of climate change. For example, using species-specific dispersal based on species’ biology, Schloss *et al*. [36] demonstrated that the extent to which mammalian taxa will be able to keep pace with climate change will depend on their dispersal abilities, with the greatest ramifications for dispersal-limited taxa in areas predicted to have rapid climate change velocities. Thus, the inclusion of more realistic dispersal and migration scenarios in SDMs is needed to improve forecasts of species’ range shifts in response to climate change.

In addition to dispersal, interactions between different species may significantly influence the ability of species to respond to climate change [6,7,12,37], yet we found that few studies incorporated these biological interactions (1618%). Further, even fewer studies incorporated both biotic interactions and dispersal simultaneously (i.e., of the 195 313 papers with SDMs including two or more species only 25 studies incorporated both biotic interactions and dispersal). We suggest that it is important to consider biotic interactions and dispersal within the same models as dispersal abilities may decouple current biotic interactions or result in new interactions in the future [14]. Despite repeated calls for the incorporation of more realistic dispersal, migration, and biologically-relevant interactions between species in SDMs over the last decade [4,10,12,16,38], our study highlights the lack of broad adoption of these practices in the scientific community. The lack of adoption of such approaches is possibly due to: 1) a lack of consensus as to what will be gained in terms of predictive ability by incorporating greater biological complexity [8,39,40]; 2) the lack of data on the biology of species replicated sufficiently across spatial scales [10]; or 3) methodological issues (but, see [41] detailing methodological advances for biotic interactions).

There were some notable biases in the SDMs (Fig 4), which may have consequences for our understanding of how species respond to climate change (e.g.,[42]). We found a paucity of studies in the Southern Hemisphere and Asia, as well as in freshwater and marine environments, even though the effects of climate change may be more severe for taxa inhabiting these ecosystems compared to the more well-studied terrestrial systems (e.g., [43,44]). Additionally, publications were predominantly focused on plants, birds, and mammals, with little coverage of groups such as fishes and herpetofauna. We hypothesize that this taxonomic and geographic bias relates to the long history of studies of terrestrial organisms in Europe and North America, with better knowledge of species distributions and biology (e.g., Atlas Florae Europaceae, NatureServe’s Mammals of the Western Hemisphere). We suggest that better knowledge on the distribution and biology of understudied organisms and regions will lead to greater usage in SDMs, improving our understanding of how species with different life histories, dispersal strategies, and biotic interactions will respond to climate change [45,46].

The majority of publications either used no climate change forecast from a GCM at all or only a single GCM forecast to simulate future conditions (Fig 5). In the case of the former, this suggests that there are some missed opportunities to understand the responses of different taxa to climate change given that models for species’ current distributions have already been built. In the case of the latter, the use of multiple climate models could improve the accuracy and realism of forecasts by accounting for variability in climate predictions between models [47].

The spatial grain of studies ranged significantly (Fig 5), even for organisms with similar modes of disperal: for instance, tree species in Europe, North America, and Asia were modeled at spatial grains ranging from ≤1km^2^ to 2,500 km^2^. Although different studies incorporate different data and objectives, our analysis suggests that consideration of species biology (e.g., dispersal ability, range size) must be taken into account when building SDMs. Studies using SDMs should explicitly report the grain size, extent, and model results with and without biological complexity (e.g., dispersal, biotic interactions) and when possible report the sensitivity of model outputs to spatial grain and extent. To this end, there is value in developing clearer guidelines for null modeling frameworks for SDMs. For instance, as more biological processes are built into SDMs, models without the added biological processes could be considered ‘null’ models.

Given that many aspects of a species’ biology (e.g., dispersal capacity, biotic interactions) are scale dependent [18–21], it is critical to consider how spatial scaling influences model predictions as biological realism is incorporated. For dispersal, we found that the effect of including simple measures of dispersal (e.g., no versus full dispersal) is not contingent on grain or extent. We found that many of the studies including biotic interactions or both biotic interactions and dispersal together did not include comparisons of model outputs with versus without the additional biological process, thus our inferences about the effect of spatial grain or extent on model outputs are conservative due to a limited sample size for the comparison. Many studies incorporating biotic interactions also only report on range changes for one species in a pairwise species interaction, which does not lend well to traditional meta-analytic approaches that account for the variance of a study’s effect size [48].

In addition to biological processes, scale dependence arising from purely statistical artifacts can also influence the outcomes of SDMs, with both grain size and extent affecting model performance [22–25]. Thus, we were surprised that grain and extent did not have more significant effects on range sizes. Independent of dispersal or biotic interactions, finer grained SDMs can have better model performance than coarser grained SDMs because spatial environmental information is lost as coarser grains homogenize landscapes [49,50], but data errors exert a stronger influence in fine grained SDMs [51]. In addition, the variation in spatial configuration of species ranges can contribute to grain size affecting results [52]. With regard to extent, increasing the spatial extent tends to improve SDM predictive power due to the incorporation of additional environmental information from surrounding areas [53]. While we examined a broader range of spatial scales than previous studies [23,49], it is important to note that our analysis does not separate the effects of biological processes from statistical scaling effects.

## Conclusion

Though much progress has been made on predicting the response of species to climate change, we have identified some critical areas of future research and guidelines for best practices in both generating and reporting these results. We synthesized data from the literature to show that spatial grain and extent do not influence outputs of SDMs that incorporate dispersal and biotic interactions. However, this result is based on a limited number of studies because the majority of studies did not report how changes in range sizes differed between models with and without dispersal or biotic interactions incorporated. Future studies should be sure to report such differences as biological processes are added into SDMs to ensure that we know what we are gaining by making models less parsimonious.

## Supporting information

S1 File Citation information for papers included in the literature review. (XLSX)

S1 Table Summary of data collected on each paper for each objective during the literature review. (DOCX)

S2 File Data collected on each paper for the objectives outlined in S2 File. (XLSX)

S3 File Dispersal and biotic interactions analyses data. (XLSX)

S2 Table PRISMA checklist. (DOC)

S1 Fig Scatterplots depicting the relationship between spatial grains and spatial extents of studies in S1 Table and S3 File. (DOCX)

## References

[1] Walther G-R, Post E, Convey P, Menzel A, Parmesan C, Beebee TJC, et al Ecological responses to recent climate change. Nature. 2002 3 28;416(6879):389–95. doi: 10.1038/416389a 11919621 10.1038/416389a

[2] Stocker T. Climate change 2013: the physical science basis: Working Group I contribution to the Fifth Assessment Report of the Intergovernmental Panel on Climate Change. Cambridge University Press; 2014.

[3] Guisan A, Zimmerman N. Predictive habitat distribution models in ecology. Ecol Model. 2000;135(2):147–86.

[4] Elith J, Leathwick JR. Species Distribution Models: Ecological Explanation and Prediction Across Space and Time. Annu Rev Ecol Evol Syst. 2009;40:677–97.

[5] Franklin J. Mapping species distributions: spatial inference and prediction. Cambridge University Press; 2010.

[6] Araújo MB, Luoto M. The Importance of Biotic Interactions for Modelling Species Distributions under Climate Change. Glob Ecol Biogeogr. 2007;16(6):743–53.

[7] Zarnetske PL, Skelly DK, Urban MC. Biotic Multipliers of Climate Change. Science. 2012 6 22;336(6088):1516–8. doi: 10.1126/science.1222732 22723403 10.1126/science.1222732

[8] Urban MC, Zarnetske PL, Skelly DK. Moving forward: dispersal and species interactions determine biotic responses to climate change. Ann N Y Acad Sci. 2013 9 1;1297(1):44–60. 23819864 10.1111/nyas.12184

[9] Wisz MS, Pottier J, Kissling WD, Pellissier L, Lenoir J, Damgaard CF, et al The role of biotic interactions in shaping distributions and realised assemblages of species: implications for species distribution modelling. Biol Rev. 2013 2 1;88(1):15–30. doi: 10.1111/j.1469-185X.2012.00235.x 22686347 10.1111/j.1469-185X.2012.00235.x PMC3561684

[10] Evans M, Merow C, Record S, McMahon S, Enquist B. Towards process based range modeling of many species. Trends Ecol Evol. 31(11):860–71. doi: 10.1016/j.tree.2016.08.005 27663835 10.1016/j.tree.2016.08.005

[11] Brooker R, Travis J, Clark E, Dytham C. Modelling species’ range shifts in a changing climate: the impacts of biotic interactions, dispersal distance and the rate of climate change. J Theor Biol. 245(1):59–65. doi: 10.1016/j.jtbi.2006.09.033 17087974 10.1016/j.jtbi.2006.09.033

[12] Gilman SE, Urban MC, Tewksbury J, Gilchrist GW, Holt RD. A framework for community interactions under climate change. Trends Ecol Evol. 2010 6;25(6):325–31. doi: 10.1016/j.tree.2010.03.002 20392517 10.1016/j.tree.2010.03.002

[13] Blois JL, Zarnetske PL, Fitzpatrick MC, Finnegan S. Climate Change and the Past, Present, and Future of Biotic Interactions. Science. 2013 8 2;341(6145):499–504. doi: 10.1126/science.1237184 23908227 10.1126/science.1237184

[14] Hellmann JJ, Prior KM, Pelini SL. The influence of species interactions on geographic range change under climate change. Ann N Y Acad Sci. 2012 2 1;1249(1):18–28. 22329888 10.1111/j.1749-6632.2011.06410.x

[15] Shmida A, Wilson MV. Biological Determinants of Species Diversity. J Biogeogr. 1985 1 1;12(1):1–20.

[16] Pearson RG, Dawson TP. Predicting the Impacts of Climate Change on the Distribution of Species: Are Bioclimate Envelope Models Useful? Glob Ecol Biogeogr. 2003;12(5):361–71.

[17] McGill BJ. Matters of Scale. Science. 2010;328(5978):575–6. doi: 10.1126/science.1188528 20431001 10.1126/science.1188528

[18] Trakhtenbrot A, Nathan R, Perry G, Richardson DM. The importance of long-distance dispersal in biodiversity conservation. Divers Distrib. 2005 3 1;11(2):173–81.

[19] Thomson FJ, Moles AT, Auld TD, Kingsford RT. Seed dispersal distance is more strongly correlated with plant height than with seed mass. J Ecol. 2011;99(6):1299–307.

[20] Araújo MB, Rozenfeld A. The geographic scaling of biotic interactions. Ecography. 2013 12 1;no-no.

[21] Belmaker J, Zarnetske P, Tuanmu M-N, Zonneveld S, Record S, Strecker A, et al Empirical evidence for the scale dependence of biotic interactions. Glob Ecol Biogeogr. 2015 7 1;24(7):750–61.

[22] Wiens JA. Spatial Scaling in Ecology. Funct Ecol. 1989;3(4):385–97.

[23] Guisan A, Graham CH, Elith J, Huettmann F, Group NSDM. Sensitivity of Predictive Species Distribution Models to Change in Grain Size. Divers Distrib. 2007;13(3):332–40.

[24] Seo C, Thorne JH, Hannah L, Thuiller W. Scale effects in species distribution models: implications for conservation planning under climate change. Biol Lett. 2009 2 23;5(1):39–43. doi: 10.1098/rsbl.2008.0476 18986960 10.1098/rsbl.2008.0476 PMC2657743

[25] Austin MP, Van Niel KP. GUEST EDITORIAL: Improving species distribution models for climate change studies: variable selection and scale. J Biogeogr. 2011;38(1):1–8.

[26] Record S, Fitzpatrick MC, Finley AO, Veloz S, Ellison AM. Should species distribution models account for spatial autocorrelation? A test of model projections across eight millennia of climate change. Glob Ecol Biogeogr. 2013 6 1;22(6):760–71.

[27] Bateman BL, Murphy HT, Reside AE, Mokany K, VanDerWal J. Appropriateness of full-, partial- and no-dispersal scenarios in climate change impact modelling. Divers Distrib. 2013 10 1;19(10):1224–34.

[28] Merow C, Smith MJ, Edwards TC, Guisan A, McMahon SM, Normand S, et al What do we gain from simplicity versus complexity in species distribution models? Ecography. 2014 12 1;37(12):1267–81.

[29] Nobis MP, Normand S. KISSMig–a simple model for R to account for limited migration in analyses of species distributions. Ecography. 2014 12 1;37(12):1282–7.

[30] Engler R, Guisan A. MigClim: Predicting plant distribution and dispersal in a changing climate. Divers Distrib. 2009 7;15(4):590–601.

[31] Fitzpatrick MC, Preisser EL, Porter A, Elkinton J, Ellison AM. Modeling range dynamics in heterogeneous landscapes: invasion of the hemlock woolly adelgid in eastern North America. Ecol Appl. 2012;22(2):472–86. 22611848 10.1890/11-0009.1

[32] Moher D, Liberati A, Tetzlaff J, Altman D, The PRISMA Group. Preferred reporting items for systematic reviews and meta-analyses: the PRISMA statement. PLoS Med. 6(7):e1000097 doi: 10.1371/journal.pmed.1000097 19621072 10.1371/journal.pmed.1000097 PMC2707599

[33] Romo H, García-Barros E, Márquez AL, Moreno JC, Real R. Effects of climate change on the distribution of ecologically interacting species: butterflies and their main food plants in Spain. Ecography. 2014 7;1063–1072n/a–n/a.

[34] Vittoz P. Seed dispersal distances: a typology based on dispersal modes and plant traits. Bot Helvetica. 117(2):109–24.

[35] Whitmee S, Orme CDL. Predicting dispersal distance in mammals: a trait-based approach. J Anim Ecol. 2013 1 1;82(1):211–21. doi: 10.1111/j.1365-2656.2012.02030.x 22924343 10.1111/j.1365-2656.2012.02030.x

[36] Schloss CA, Nuñez TA, Lawler JJ. Dispersal will limit ability of mammals to track climate change in the Western Hemisphere. Proc Natl Acad Sci U S A. 2012;109(22):8606–11. doi: 10.1073/pnas.1116791109 22586104 10.1073/pnas.1116791109 PMC3365214

[37] Post E. Ecology of climate change: the importance of biotic interactions. Princeton University Press;

[38] Guisan A, Thuiller W. Predicting species distribution: offering more than simple habitat models. Ecol Lett. 2005 9 1;8(9):993–1009. 10.1111/j.1461-0248.2005.00792.x 34517687

[39] Godsoe W, Harmon LJ. How do species interactions affect species distribution models? Ecography. 2012 9 1;35(9):811–20.

[40] Urban MC, Tewksbury JJ, Sheldon KS. On a collision course: competition and dispersal differences create no-analogue communities and cause extinctions during climate change. Proc Biol Sci. 2012;279(1735):2072–80. doi: 10.1098/rspb.2011.2367 22217718 10.1098/rspb.2011.2367 PMC3311897

[41] Kissling WD, Dormann CF, Groeneveld J, Hickler T, Kühn I, McInerny GJ, et al Towards novel approaches to modelling biotic interactions in multispecies assemblages at large spatial extents. J Biogeogr. 2012 12 1;39(12):2163–78.

[42] Lenoir J, Svenning J-C. Climate-related range shifts–a global multidimensional synthesis and new research directions. Ecography. 2015 1 1;38(1):15–28.

[43] Ricciardi A, Rasmussen JB. Extinction Rates of North American Freshwater Fauna. Conserv Biol. 1999;13(5):1220–2.

[44] Colwell RK, Brehm G, Cardelús CL, Gilman AC, Longino JT. Global Warming, Elevational Range Shifts, and Lowland Biotic Attrition in the Wet Tropics. Science. 2008;322(5899):258–61. doi: 10.1126/science.1162547 18845754 10.1126/science.1162547

[45] Feeley KJ, Silman MR. The data void in modeling current and future distributions of tropical species. Glob Change Biol. 2011 1 1;17(1):626–30.

[46] Beck J, Ballesteros-Mejia L, Buchmann CM, Dengler J, Fritz SA, Gruber B, et al What’s on the horizon for macroecology? Ecography. 2012 8 1;35(8):673–83.

[47] Araújo MB, New M. Ensemble forecasting of species distributions. Trends Ecol Evol. 2007 1;22(1):42–7. doi: 10.1016/j.tree.2006.09.010 17011070 10.1016/j.tree.2006.09.010

[48] Hedges LV, Gurevitch J, Curtis PS. The Meta-Analysis of Response Ratios in Experimental Ecology. Ecology. 1999;80(4):1150–6.

[49] Gottschalk TK, Aue B, Hotes S, Ekschmitt K. Influence of grain size on species–habitat models. Ecol Model. 2011 9 24;222(18):3403–12.

[50] Randin CF, Engler R, Normand S, Zappa M, Zimmermann NE, Pearman PB, et al Climate change and plant distribution: local models predict high-elevation persistence. Glob Change Biol. 2009 6 1;15(6):1557–69.

[51] Hanberry BB. Finer grain size increases effects of error and changes influence of environmental predictors on species distribution models. Ecol Inform. 2013 5;15:8–13.

[52] Lauzeral C, Grenouillet G, Brosse S. Spatial range shape drives the grain size effects in species distribution models. Ecography. 2013 7 1;36(7):778–87.

[53] Song W, Kim E, Lee D, Lee M, Jeon S-W. The sensitivity of species distribution modeling to scale differences. Ecol Model. 2013 1 10;248:113–8.

