# Supplementary material for: Does scale matter? A systematic review of incorporating biological realism when predicting changes in species distributions
Source: PLoS One. 2018 Apr 13;13(4):e0194650. doi: 10.1371/journal.pone.0194650 (PMC5898710; doi:10.1371/journal.pone.0194650)
Supplement: S1 Table — (DOCX) [file pone.0194650.s002.docx]

**Supporting Information S2**

**Table S2-1.** Summary of data collected on each paper for each objective during the literature review.

| **Objective 1: How does the scaling of biological processes change SDM predictions?** | **Objective 2: What is the current state of SDMs incorporating biotic interactions, dispersal, and interactions between the two?** |
| --- | --- |
| - Spatial extent of SDM (km^2^) | - Number of taxa modeled |
| - Spatial grain of SDM (km^2^) | - Type(s) and number of taxa modeled |
| - Scale of SDM (regional, continental, global) | - Geographic location(s) of study |
| - Biotic interaction included in SDM? (binary response) | - Realm of study (i.e., terrestrial, marine, aquatic) |
| - If SDM incorporates biotic interactions and predicted range sizes were compared between models, what was the direction and amount of change in range size with biotic interactions? | - Whether or not multiple GCMs were used if the models were projected to a time point other than current. If so, how many climate models were used? |
| - Dispersal included in SDM? (binary response) - If SDM incorporates dispersal, was taxon-specific information used? |  |
| - If SDM incorporates dispersal and predicted range sizes were compared between models, what was the direction and amount of change in range size with dispersal? |  |
