# Supplementary material for: Does scale matter? A systematic review of incorporating biological realism when predicting changes in species distributions
Source: PLoS One. 2018 Apr 13;13(4):e0194650. doi: 10.1371/journal.pone.0194650 (PMC5898710; doi:10.1371/journal.pone.0194650)
Supplement: S1 Fig — (DOCX) [file pone.0194650.s006.docx]

**Appendix S6.** Scatterplots depicting the relationship between spatial grains and spatial extents of studies in S3 and S4.


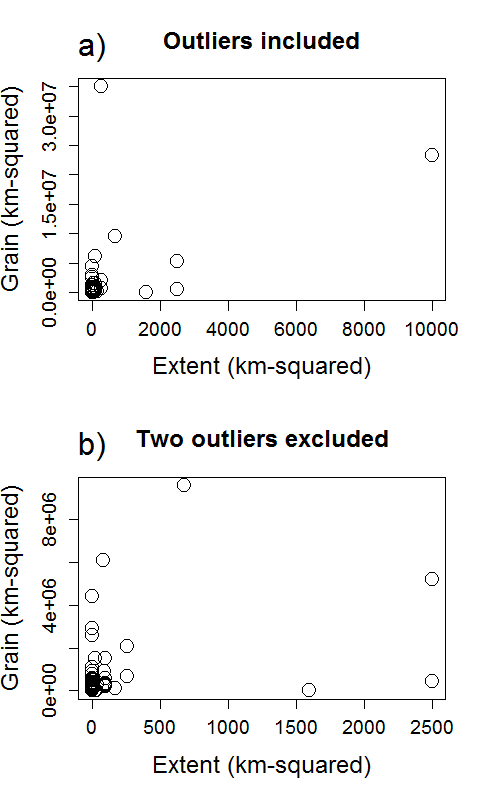


**Figure 1.** a) Scatterplots showing the relationship between spatial grain and spatial extent for all reviewed studies (listed in App. S3) that reported both spatial grain and extent (*ρ* =0.53). b) Scatterplots showing the relationship between spatial grain and spatial extent for all reviewed studies reporting both spatial grain and extent, but excluding two outliers – one with high spatial grain and one with high spatial extent (*ρ* =0.34).


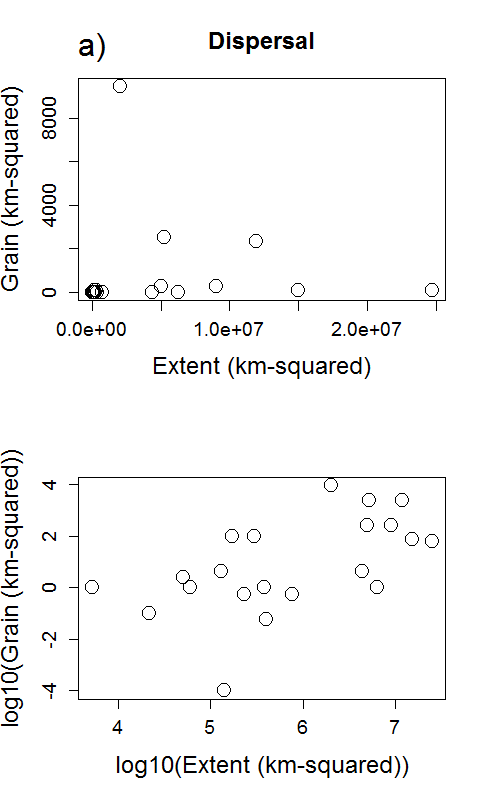


**Figure 2.** a) Scatterplots showing the relationship between spatial grain and spatial extent for all studies included in the dispersal mixed effects model analysis (listed in App. S4) (*ρ* = 0.02). b) Scatterplots showing the relationship between log10(spatial grain) and log10(spatial extent) for all studies included in the dispersal mixed effects model analysis (listed in App. S4) (*ρ* = 0.56).


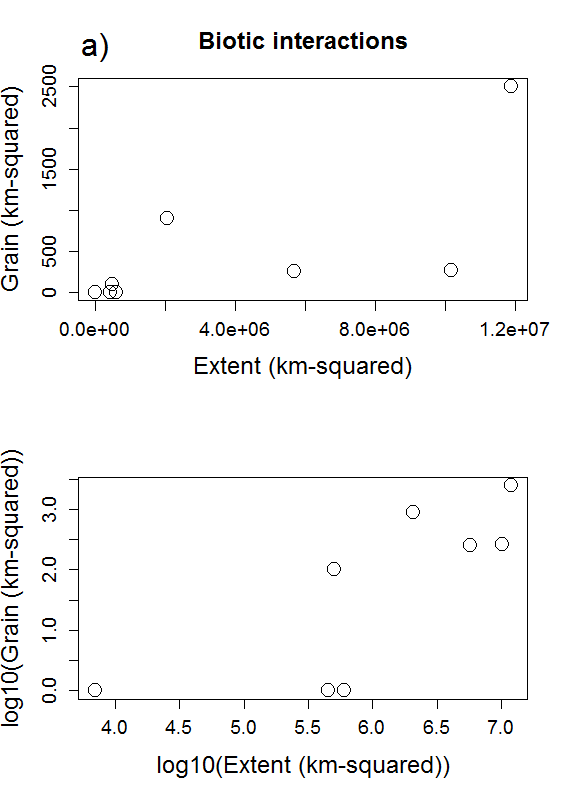


**Figure 3.** a) Scatterplots showing the relationship between spatial grain and spatial extent for all studies included in the biotic interactions mixed effects model analysis (listed in App. S4) (*ρ* = 0.71). b) Scatterplots showing the relationship between log10(spatial grain) and log10(spatial extent) for all studies included in the dispersal mixed effects model analysis (listed in App. S4) (*ρ* = 0.73).
